# Partial trisomy 16q21➔qter due to an unbalanced segregation of a maternally inherited balanced translocation 46,XX,t(15;16)(p13;q21): a case report and review of literature

**DOI:** 10.1186/s12887-017-0980-z

**Published:** 2018-01-08

**Authors:** R. Mishra, C. S. Paththinige, N. D. Sirisena, S. Nanayakkara, U. G. I. U. Kariyawasam, V. H. W. Dissanayake

**Affiliations:** 1 Human Genetics Unit, Faculty of Medicine, University of Colombo, Kynsey Road, Colombo, 00800 Sri Lanka; 2 Castle Street Hospital for Women, Colombo, 00800 Sri Lanka; 3 Civil Service Hospital, Minbhawan Marg, Minbhawan, Kathmandu, 44600 Nepal; 4 Faculty of Medicine and Allied Sciences, Rajarata University of Sri Lanka, Saliyapura, Anuradhapura, 50008 Sri Lanka

**Keywords:** Partial trisomy 16q, Congenital heart disease, Anteriorly placed anus, Chromosomal translocation

## Abstract

**Background:**

Partial trisomy is often the result of an unbalanced segregation of a parental balanced translocation. Partial trisomy16q is characterized by a common, yet non-specific group of craniofacial dysmorphic features, and systemic malformations with limited post-natal survival. Most of the cases of partial trisomy 16q described in the scientific literature have reported only one, or less frequently two cardiac defects in the affected babies. Herein, we report a case of partial trisomy 16q21➔qter with multiple and complex cardiac defects that have not previously been reported in association with this condition.

**Case presentation:**

We report the phenotypic and cytogenetic features of a Sri Lankan female infant with partial trisomy 16q21➔qter. The baby had a triangular face with downslanting eyes, low set ears and a cleft palate. Systemic abnormalities included multiple cardiac defects, namely double outlet right ventricle, ostium secundum atrial septal defect, mild pulmonary stenosis, small patent ductus arteriosus, and bilateral superior vena cavae. An anteriorly placed anus was also observed. The proband was trisomic for 16q21➔qter chromosomal region with a karyotype, 46,XX,der(15)t(15;16)(p13;q21)mat. The chromosomal anomaly was the result of an unbalanced segregation of a maternal balanced translocation; 46,XX,t(15;16)(p13;q21). Partial trisomy 16q was established by fluorescence in-situ hybridization analysis.

**Conclusions:**

The craniofacial dysmorphic features and the presence of cardiac and anorectal malformation in the proband are consistent with the phenotypic spectrum of partial trisomy 16q reported in the scientific literature. More proximal breakpoints in chromosome 16q are known to be associated with multiple cardiac abnormalities and poor long-term survival of affected cases. This report presents a unique case with multiple, complex cardiac defects that have not previously been described in association with a distal breakpoint in 16q. These findings have important diagnostic and prognostic implications.

## Background

Trisomy 16 is reported to be the most frequent trisomy detected in first trimester spontaneous abortions [1, 2]. Few cases have also been reported in second and third trimesters. Among them, some are confined to the placenta [3]. The range of trisomy 16 varies from full trisomy [4, 5], to mosaics [6–8], to partial trisomy of 16p [9] or 16q [10]. Full trisomy 16 almost always leads to spontaneous abortion and most of the babies with partial trisomy 16 suffer from congenital abnormalities with limited post-natal survival. Partial trisomy 16 often results from an unbalanced segregation of a parental balanced translocation. Most of these cases were reported in newborns. In few cases, prenatal ultrasonography showed gross fetal anomalies and prenatal diagnosis of partial trisomy 16 was done by cytogenetic studies [11]. The commonly reported clinical features of partial trisomy of the long arm of chromosome 16 were low birth weight, hypotonia, failure to thrive, psychomotor retardation, periorbital oedema, high prominent forehead, microcephaly, low set ears, flat nasal bridge, small and downslanting palpebral fissures, micrognathia, hypertelorism, long philtrum, and posterior cleft palate. Systemic abnormalities such as congenital heart defects, renal abnormalities, lung abnormalities and gall bladder agenesis were also reported [12]. More specifically, in patients with trisomy of the distal segment of the long arm of chromosome 16, the cardiac defects were uncommon [13–17]. These patients survived longer, except one patient [14] who died 22 days after birth despite the absence of cardiac defects or other systemic abnormalities. Anorectal anomalies [15] and skeletal defects [13, 14, 17] were also reported in some of the cases with terminal 16q duplication. Common dysmorphic features observed in these patients include small and downslanting palpebral features, hypertelorism, low set ears and flat nasal bridge. [13–17]. The absence of a specific group of dysmorphic features and systemic abnormalities makes the clinical diagnosis of this condition difficult. Herein, we present the case of an infant with a karyotype of 46,XX,der(15)t(15;16)(p13;q21)mat with craniofacial dysmorphism, an anteriorly placed anus, and multiple congenital cardiac defects that have not previously been reported in association with this condition.

## Case presentation

The proband was referred for genetic evaluation due to multiple congenital abnormalities at the age of 24 days. She was born to a non-consanguineous couple, a 34-year-old father and a 32-year-old mother who gave a history of a previous first trimester miscarriage and a neonatal death soon after birth due to congenital cardiac defects. Karyotyping and autopsy of the deceased neonate were not done. No further clinical records of the previous pregnancies were available.

There were no antenatal complications during the third pregnancy. Premature rupture of membranes occurred at 33 weeks of gestation resulting in pre-term delivery. The proband’s weight was below the 5th centile (1230 g) while the length (42 cm) and the head circumference (28 cm) were below the 50th percentile at birth. Physical examination showed a triangular face with small and downslanting palpebral fissures, low set ears, cleft palate and an anteriorly placed anus. She had a murmur on auscultation. Hypotonia was noted on neurological examination. 2D–echocardiography showed a double outlet right ventricle, ostium secundum atrial septal defect, mild pulmonary stenosis, small patent ductus arteriosus, and bilateral superior vena cavae. Global developmental delay and failure to thrive were noted on follow up and physiotherapy was arranged. She died at the age of 10 months due to complications arising from multiple cardiac defects.

**Fig. 1:**
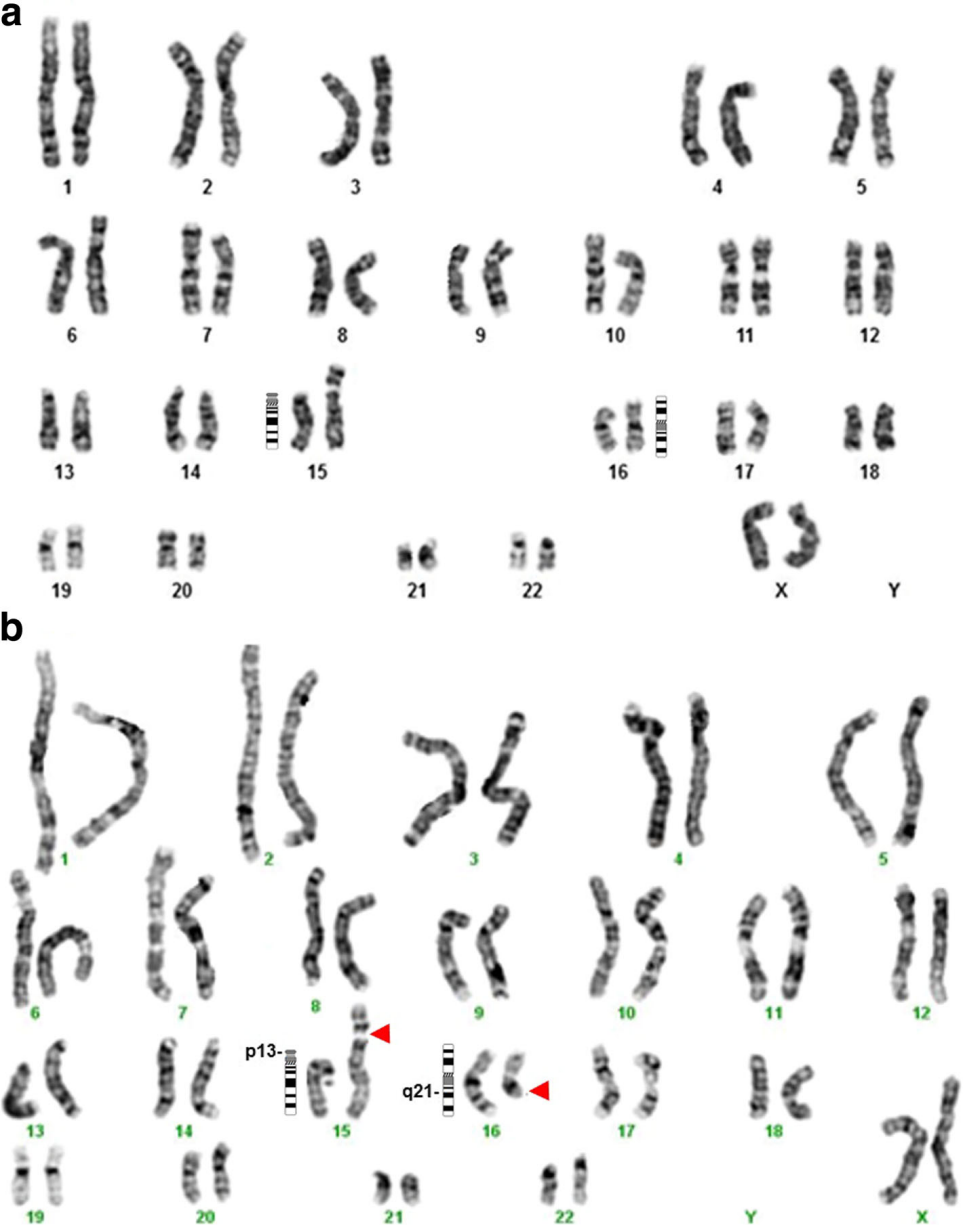
Karyograms of the proband and her mother. **a** The proband’s karyogram showing the karyotype, 46,XX,der(15)t(15;16)(p13;q21)mat. **b** The proband’s mother’s karyogram showing the karyotype, 46,XX,t(15;16)(p13;q21)

Blood from the proband, and her parents were obtained for karyotyping after obtaining written informed consent. Initially the proband was tested. The parents were tested after detection of the derivative chromosome 15 in the proband, to find out whether one of them was a balanced translocation carrier. Metaphase cell preparation was obtained from 72 h’ culture of phytohaemagglutinin stimulated lymphocytes in PB-MAX culture media [Invitrogen]. The cells were analysed after GTG banding. The proband’s karyotype was 46,XX,der(15)t(15;16)(p13;q21)mat (Fig. 1a). Her mother’s karyotype was 46,XX,t(15;16)(p13;q21) (Fig. 1b) and her father’s karyotype was 46,XY. Thus, the proband was trisomic for 16q21➔qter due to an unbalanced segregation of a maternally inherited balanced translocation.

**Fig. 2:**
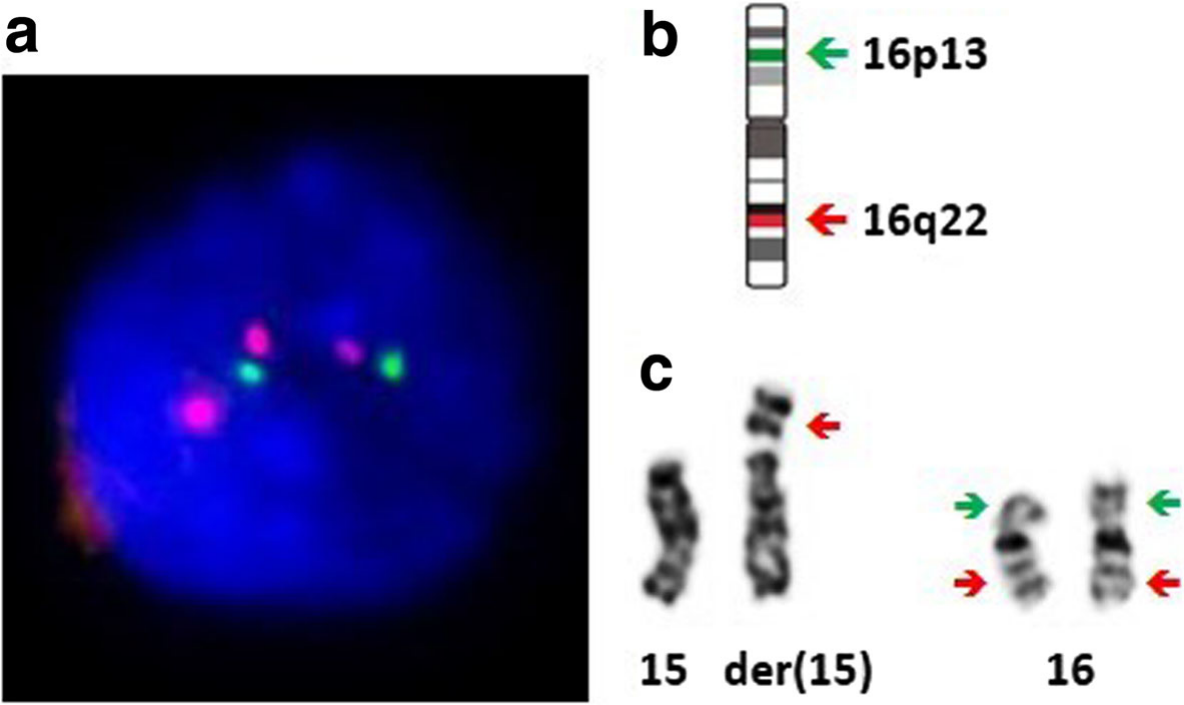
FISH analysis on interphase chromosomes of the proband. **a**) FISH image of the proband showing two hybridization signals on 16p13 region (green) and three hybridization signals on 16q22 region (red). **b**) Ideogram of the chromosome 16 indicating the hybridization points of the FISH probes, 16p13 including the *MYH11* gene (spectrum green) and 16q22 including the *CBFB* gene (spectrum red) (Metasystems, Altlussheim, Germany). **c**) Partial karyogram of the proband indicating the hybridization points in the two chromosomes 16 and the derivative chromosome 15

Further analysis of the structural chromosomal rearrangement in the proband was done using fluorescent in-situ hybridization (FISH) technique on interphase chromosomes using locus specific probes for 16p13 including the *MYH11* gene (spectrum green) and 16q22 including the *CBFB* gene (spectrum red) [Metasystems, Altlussheim, Germany]. FISH analysis showed normal (two) hybridization signals for 16p13. A total of three hybridization signals for 16q22 region was seen in each cell in interphase preparation, indicating that the breakpoint in the chromosome 16 is proximal to the *CBFB* gene (16q22) locus (Fig. 2). This finding supported the karyotype result which showed trisomy for 16q21➔qter. Further demarcation of the chromosomal breakpoint and delineation of the duplicated segment of the long arm of chromosome 16 could not be done, because the required technology such as microarray based comparative genomic hybridization (array-CGH) has not yet been established at our centre.

## Discussion

The cytogenetic studies of the proband showed trisomy 16q21➔qter. The distal fragile site of the long arm of chromosome 16 is commonly reported to be located between 16q21 and 16q22, as observed in our case [18–20]. Table 1 describes the cytogenetic and phenotypic data of the proband in comparison with previously reported cases with trisomy 16q [10, 13–17, 21–25]. Most of the affected children had abnormal, yet non-specific craniofacial features with flat nasal bridge, small and downslanting palpebral fissures and low set ears which were also observed in the present case. High arched palate [15, 17, 23, 24] and ear malformations [14, 16, 17, 23, 24] were commonly reported in previous cases. The proband also had a cleft palate which was reported in only one previous case of trisomy 16q [24]. Anorectal malformations, which were present in the proband were also reported in two previous cases in the published literature [15, 24]. Skeletal abnormalities and limb defects, although reported commonly in previous reports, were not observed in the proband. More notably, all the male children in the cases reviewed were found to have hypoplastic or ambiguous external genitalia [10, 13, 14, 23, 25].

**Table 1:** Clinical features of the present case and previously reported cases of trisomy 16q

	Present case	Francke (1972) [21]	Balestrazzi et al. (1979) [13]	Ridler and Mckeown (1979) [22]	Garau et al. (1980) [14]	Nevin et al. (1983) [23]		Davison and Beesley (1984) [10]		Hatanaka et al. (1984) [24]	Houlston et al. (1994) [15]	Savary et al. (1991) [16]	Paladini et al. (1999) [25]	de Carvalho et al. (2010) [17]
Trisomy	16q21 ➔qter	16q?	16q21➔qter	16q11➔qter	16q21➔qter	16q11➔qter	16q11➔qter	16q13➔ qter	16q13➔qter	16q13➔qter	16q22➔qter	16q23 ➔qter	16q12.1➔qter	16q21➔qter
Parental translocation	t(15;16) (p13;q21) mat	t(16q;22q) pat	t(16;22) (q21;p12) mat	t(15;16) (p11;q11) mat	t(16;18) (q21;p11.2) pat	t(15;16) (p12;q11) mat	t(15;16) (p12;q11) mat	t(16;20) (q13;p13) pat	t(16;20) (q13;p13) pat	t(11;16) (q25;q13) pat	[t(15;16) (q26.1;q22)- de novo duplication]	t(13;16) (p12;q23) mat	T(16;20) (q12.1;p13) pat	t(4;16) (q32;q21) mat
Sex	Female	Female	Male	Female	Male	Male	Male	Female	Male	Female	Female	Female	Male	Female
LBW	+	+		+	+	+	+	+	+	+	+		+	+
Skull abnormalities			Asymmetry				Brachycephaly			Dolicocephaly				Microcephaly, Craniosynostosis
Prominent/high forehead		+	+		+	+		+	+		+		+	
Abnormal face	Triangular			Narrow		Triangular	Triangular		Elphin					
Micrognathia			+	+		+	+	+		+	+		+	+
Hypertelorism			+		+							+	+	
Palpebral fissures	Small Down-slant		Small Down-slant		Small	Small Down-slant	Small Down-slant			Small	Small Down-slant	Small Upslanting		Small
Flat nasal bridge		+	+	+	+			+	+	+		+	+	+
Abnormal nose			Bulbous	Beaked			Bulbous				Choanal atresia			
Ear abnormality	Low set		Low set	Low set, large	Low set, malformed	Malformed	Malformed	Low set	Low set	Low set, malformed	Low set	Malformed	Low set	Low set, malformed
Thin upper lip			+			+				+	+			
Abnormalities in palate	Cleft palate					High arched	High arched			High arched, cleft +	High arched			High arched
Congenital cardiac defects	DORV, OS- ASD, PS, PAD, B/L SVC	PAD		ASD		PDA		VSD	VSD	ASD, PAD			ASD, VSD, CoA	[Aneurysm of interatrial septum]
Abnormal genitalia			Congenital hydrocele Hypoplastic penis, scrotum and testis		Small penis, bifid scrotum, ? undescended R/testis	Small penis, undescended testis	Small penis, undescended testis	Prominent labia majora	Ambiguous, Hypospadias, bifid scrotum				Ambiguous genitalia	
Anorectal malformation	Anteriorly placed anus									Imperforate anus, anovestibular fistula	Anal stenosis			
Other GI malformations				Liver congestion, biliary thrombi and fibrosis						Malrotation Umbilical hernia				Umbilical hernia
Skeletal and limb defects			Pectus excavatum Kypho-scoliosis Brachydactyly Genu valgum Pes valgus Sandle gap		Elbow contracture Valgus of hands, L/S talus valgus			Syndactyly B/L feet	Rocker-bottom feet	L/S knee and hip dislocation	Small 5^th^ digit of hands			Clinodactyly L/clubfoot
Survival	10 Months	12Month	3 ½ years (alive)	12 Days	22 Days	5weeks	6 Weeks	5weeks	18 Days	6 Month	3 years (Alive)	Alive	10 Days	7 Years (Alive)

*LBW* Low birth weight, *VSD* Ventricular Septal defect, *ASD* Atrial septal defect, *PAD* Patent ductus arteriosus, *DORV* Double outlet of right ventricle, *OS* ostium secundum, *PS* Pulmonary stenosis, *B/L SVC* Bilateral superior vena cavae

Analysis of the published data showed that majority of the patients with trisomy 16q had congenital heart defects, commonly atrial septal defect (ASD), ventricular septal defect (VSD) or patent ductus arteriosus (PDA). Hatanaka et al. [24] and Paladini et al. [25] reported cases with multiple cardiac defects that included ASD and PDA in the former, and ASD, VSD and coarctation of aorta in the latter. Both these patients had a large trisomic segment of the long arm of chromosome 16 with a proximal breakpoint at q13 and q12.1, respectively. The present case had multiple and complex set of cardiac abnormalities that included ASD, PDA, pulmonary stenosis, double outlet right ventricle, and bilateral superior vena cavae. To the best of our knowledge, none of the previously reported cases of partial trisomy of the distal segment of the long arm of chromosome 16 had multiple congenital cardiac defects as seen in this case.

The trisomic segment of chromosome 16q in the present case is known to contain the *Forkhead box F1 (FOXF1)* gene at 16q24 locus. This gene plays a role in the regulation of pulmonary genes as well as embryonic development of the fetus [NCBI; Gene ID 2294]. Mutations in this gene are known to be associated with alveolar capillary dysplasia with misalignment of pulmonary vein (ACDMPV) [OMIM:601,089]. Right to left shunt via the foramen ovale or ductus arteriosus, atrial septal defect, alveolar capillary dysplasia, malposition of pulmonary vessels and neonatal pulmonary hypertension are common findings in ACDMPV [OMIM:265,380]. Another gene, Forkhead box protein C2 (*FOXC2*) is located at 16q24.1 locus [NCBI; Gene ID 2303]. Mutations in this gene cause Lymphedema-distichiasis [OMIM:602,402]. Congenital heart defects, that include tetralogy of Fallot, ventricular septal defect and patent ductus arteriosus are common findings in this syndrome [OMIM:153,400]. However, the effect of the dosage imbalance of these genes caused by partial trisomy 16q has not been clearly described.

The analysis of reported cases with trisomy 16q shows that patients with a longer trisomic segment tend to have shorter survival and a higher incidence of congenital heart defects than those who were trisomic for the distal half of the long arm [10, 13–17, 21–25]. In our case, although the duplicated segment was present in the distal half, the proband had multiple congenital cardiac defects. These findings signify the importance of this case and highlights the need for further clinical and molecular genetic evaluation of the cases with partial trisomy 16q.

## Conclusions

In conclusion, partial trisomy 16q21 → qter is characterized by a common yet nonspecific group of craniofacial dysmorphism and congenital anomalies. This report provides further evidence to highlight the association of this cytogenetic abnormality with a wide variety of congenital cardiac defects with important diagnostic and prognostic implications. Comprehensive cardiac assessment of these babies should be done early in life and prior to any corrective surgical procedures for other abnormalities, because the presence of cardiac abnormalities is known to be associated with poor survival. Additional genetic studies are required to identify the precise cytogenetic and molecular genetic defect in these patients in order to better elucidate the genotype-phenotype correlation.

## Abbreviations

ACDMPV: Alveolar capillary dysplasia with misalignment of pulmonary vein
ASD: Atrial septal defect
FISH: Fluorescence in-situ hybridization
PDA: Patent ductus arteriosus
VSD: Ventricular septal defect

## References

[1] Ljunger E, Cnattingius S, Lundin C, Annerén G (2005). Chromosomal anomalies in first-trimester miscarriages. Acta Obstet Gynecol Scand.

[2] Petracchi F, Igarzabal L, Crespo ML, Gadow E (2009). Trisomy 16 detected by first trimester screening. Prenat Diagn.

[3] Post JG, Nijhuis JG (1992). Trisomy 16 confined to the placenta. Prenat Diagn.

[4] Cusick W, Bork M, Fabri B, Benn P, Rodis JF, Buttino L (1995). Trisomy 16 fetus surviving into the second trimester. Prenat Diagn.

[5] Yancey MK, Hardin EL, Pacheco C, Kuslich CD, Donlon TA (1996). Non-mosaic trisomy 16 in a third-trimester fetus. Obstet Gynecol.

[6] Gilbertson NJ, Taylor JW, Kovar IZ (1990). Mosaic trisomy 16 in a live newborn infant. Arch Dis Child.

[7] Devi AS, Velinov M, Kamath MV, Eisenfeld L, Neu R, Ciarleglio L, Greenstein R, Benn P (1993). Variable clinical expression of mosaic trisomy 16 in the newborn infant. Am J Med Genet.

[8] Paulyson KJ, Sherer DM, Christian SL, Lewis KM, Ledbetter DH, Salafia CM, Meck JM (1996). Prenatal diagnosis of an infant with mosaic trisomy 16 of paternal origin. Prenat Diagn.

[9] Léonard C, Huret JL, Imbert MC, Lebouc Y, Selva J, Boulley AM (1992). Trisomy 16p in a liveborn offspring due to maternal translocation t(16; 21)(q11;p11) and review of the literature. Am J Med Genet.

[10] Davison EV, Beesley JR (1984). Partial trisomy 16 as a result of familial 16;20 translocation. J Med Genet.

[11] Puhl AG, Zelazny J, Galetzka D, Skala C, Frey-Mahn G, Wellek B, Koelbl H (2010). Unbalanced translocation 6p/16q (partial monosomy 6p and trisomy 16q): prenatal diagnosis and cytogenetics. Eur J Obstet Gynaecol Reprod Biol.

[12] Brisset S, Joly G, Ozilou C, Lapierre JM, Gosset P, LeLorc'h M, Raoul O, Turleau C, Vekemans M, Romana SP (2002). Molecular characterization of partial trisomy 16q24. 1-qter: clinical report and review of the literature. Am J Med Genet.

[13] Balestrazzi P, Giovannelli G, Rubini LL, Dallapiccola B (1979). Partial trisomy 16q resulting from maternal translocation. Hum Genet.

[14] Garau A, Crisponi G, Peretti D, Vanni R, Zuffardi O (1980). Trisomy 16q21→qter. Hum Genet.

[15] Houlston RS, Renshaw RM, James RS, Ironton R, Temple IK (1994). Duplication of 16q22→qter confirmed by fluorescence in situ hybridisation and molecular analysis. J Med Genet.

[16] Savary JB, Vasseur F, Manouvrier S, Daudignon A, Lemaire O, Thieuleux M, Poher M, Lequien P, Deminatti MM (1991). Trisomy 16q23→qter arising from a maternal t (13; 16)(p12;q23): case report and evidence of the reciprocal balanced maternal rearrangement by the Ag-NOR technique. Hum Genet.

[17] de Carvalho AF, da Silva Bellucco FT, dos Santos NP, Pellegrino R, de Azevedo Moreira LM, Toralles MB, Kulikowski LD, Melaragno MI (2010). Trisomy 16q21→ qter: seven-year follow-up of a girl with unusually long survival. Am J Hum Genet.

[18] Sørensen K, Nielsen J, Holm V, Haahr J (1979). Fragile site long arm chromosome 16. Hum Genet.

[19] Sutherland GR (1979). Heritable fragile sites on human chromosomes II. Distribution, phenotypic effects, and cytogenetics. Am J Hum Genet.

[20] Schmid M, Klett C, Niederhofer A (1980). Demonstration of a heritable fragile site in human chromosome 16 with distamycin a. Cytogenet Genome Res.

[21] Francke U (1972). Quinacrine mustard fluorescence of human chromosomes: characterization of unusual translocations. Am J Hum Genet.

[22] Ridler MA, McKeown JA (1979). Trisomy 16q arising from a maternal 15p; 16q translocation. J Med Genet.

[23] Nevin NC, Coffey WW, Nevin J, Reid M (1983). Partial trisomy 16q in two boys resulting from a maternal translocation, t (15; 16)(p12; q11). Clin Genet.

[24] Hatanaka K, Ozaki M, Suzuki M, Murata R, Fujita H (1984). Trisomy 16q13→ qter in an infant from at (11; 16)(q25; q13) translocation-carrier father. Hum Genet.

[25] Paladini D, D'Agostino A, Liguori M, Teodoro A, Tartaglione A, Colombari S, Martinelli P (1999). Prenatal findings in trisomy 16q of paternal origin. Prenat Diagn.

